# Electroacupuncture Attenuates High‐Fat Diet‐Exacerbated Alzheimer's Pathology by Enhancing TFEB/TFE3‐Mediated Autophagic Clearance of Tau and NLRP3 Inflammasome in 3xTg Mice

**DOI:** 10.1111/cns.70497

**Published:** 2025-07-01

**Authors:** Xiaoyan Zheng, Zhihao Luo, Jialin Zheng, Chuyu Deng, Dongmin Liu, Yiming Chen, Runjin Zhou, Jihua Zou, Guozhi Huang, Qing Zeng, Chunzhi Tang, Ju‐Xian Song

**Affiliations:** ^1^ School of Rehabilitation Sciences Southern Medical University Guangzhou China; ^2^ Medical College of Acupuncture‐Moxibustion and Rehabilitation Guangzhou University of Chinese Medicine Guangzhou China; ^3^ Department of Rehabilitation Medicine, Zhujiang Hospital Southern Medical University Guangzhou Guangdong China; ^4^ School of Pharmacy Chengdu University of Traditional Chinese Medicine Chengdu China

**Keywords:** Alzheimer's pathology, electroacupuncture, high‐fat diet, TFEB/TFE3

## Abstract

**Background:**

Alzheimer's disease (AD) pathology is increasingly linked to metabolic disturbances induced by high‐fat diets (HFD), with both autophagy‐lysosomal pathway (ALP) dysfunction and activation of the NLRP3 inflammasome emerging as key pathological mechanisms. This study introduces a novel approach using electroacupuncture (EA) to mitigate HFD‐accelerated AD pathology, focusing on the regulatory role of transcription factors TFEB and TFE3 in ALP and their potential modulation by EA.

**Methods:**

3xTg‐AD mice were fed either an HFD or a standard diet for 16 weeks, followed by EA treatment at the ST36 acupoint. AAV‐mediated hippocampal knockdown of TFEB and TFE3 was performed using shRNA constructs. Cognitive performance was evaluated with the Morris water maze (MWM) and Y‐maze tests. Molecular analyses, including immunoblotting and immunohistochemistry, were conducted to examine the impact of EA on NLRP3 inflammasome activation, ALP dynamics, and the involvement of TFEB and TFE3 in modulating these pathways within the brain.

**Results:**

HFD feeding significantly exacerbated Tau pathology and NLRP3 inflammasome activation in 3xTg‐AD mice, leading to pronounced cognitive deficits. Notably, EA‐ST36 intervention reversed these pathological effects, enhancing memory function and reducing both Tau aggregation and NLRP3 inflammasome activation. Mechanistic insights revealed that EA‐ST36 activated TFEB and TFE3 by modulating the MTOR/ULK1 signaling pathway, thereby enhancing autophagic clearance of NLRP3 inflammasome components and Tau aggregates.

**Conclusion:**

This study proposes EA‐ST36 as a promising therapeutic strategy for counteracting HFD‐induced AD pathology by activating TFEB/TFE3‐driven autophagic pathways, thereby enhancing the clearance of toxic protein aggregates and inflammasome components. These findings advance our understanding of EA's molecular mechanisms in AD therapy, offering a novel, nonpharmacological approach for mitigating diet‐induced exacerbation of neurodegenerative processes.

## Graphical abstract

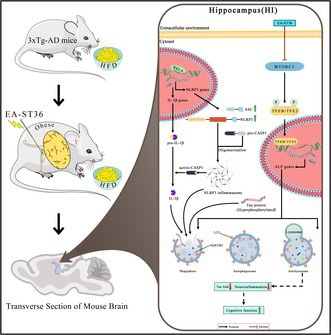

**Abbreviations**
AD: Alzheimer disease
AKT1: thymoma viral proto‐oncogene 1
ALP: autophagy‐lysosomal pathway
APP: amyloid beta (A4) precursor protein
Aβ: β‐amyloid
CTFs: C‐terminal fragments
CTSD: cathepsin D
EA: electroacupuncture
EA‐ST36: electroacupuncture at ST36 acupoint
HI: hippocampus
LAMP1: lysosomal‐associated membrane protein 1
MAP1LC3B/LC3B: microtubule‐associated protein 1 light chain 3 beta
MAPT: micro‐tubule‐associated protein tau
MTORC1: mechanistic target of rapamycin kinase complex 1
MWM: Morris water maze
MWM: Morris water maze
NFT: neurofibrillary tangles
PFC: prefrontal cortex
PSEN1: presenilin 1
SQSTM1/p62: sequestosome 1
TFEB: transcription factor EB

## 1 Introduction

Alzheimer's disease (AD) affects over 55 million people globally, with projections exceeding 139 million by 2050 due to aging populations and lifestyle factors such as obesity [1]. AD is the leading cause of dementia worldwide, characterized by progressive cognitive decline and distinct neuropathological features, such as amyloid‐β (Aβ) plaque accumulation and neurofibrillary tangles resulting from hyperphosphorylated tau proteins. Despite decades of intensive research, no therapeutic intervention has proven effective in reversing or significantly slowing the progression of ad [2]. While amyloid and tau pathologies remain central to our understanding of AD, recent findings highlight additional mechanisms, including chronic neuroinflammation and metabolic dysregulation, as critical contributors to AD pathology. Increasing evidence further implicates diet and nutrition as modifiable factors influencing AD onset and progression [3]. Specifically, a high‐fat diet (HFD) has been shown to induce metabolic disturbances, including obesity, systemic inflammation, dyslipidemia, insulin resistance, and hyperglycemia [4], which collectively may exacerbate neurodegenerative processes associated with AD. Experimental studies using triple‐transgenic (3xTg‐AD) mouse models have demonstrated that HFD feeding exacerbates amyloid deposition, tau pathology, and impairments in cognitive function [5, 6, 7, 8]. Although the impact of HFD on Aβ aggregation is well‐documented [9], the influence on tau pathology remains less understood, warranting further investigation into the molecular pathways by which HFD may accelerate AD pathology.

Recent studies underscore the pivotal role of the NLRP3 inflammasome in the pathophysiology of AD [10, 11]. The NLRP3/IL‐1β signaling axis is increasingly recognized as a critical link between metabolic disorders and cognitive decline. Aberrant activation of the NLRP3 inflammasome drives neuroinflammatory responses, and inhibition of inflammasome components—such as NLRP3 itself, ASC, or IL‐1β—has been shown to mitigate inflammatory responses and improve cognitive outcomes in animal models of ad [ 12]. Moreover, NLRP3 activation is closely associated with obesity‐related inflammation, insulin resistance, and type 2 diabetes [13, 14]. Inhibiting the NLRP3 inflammasome has been observed to enhance metabolic health and lifespan in HFD‐induced obesity models [15], positioning NLRP3 as a promising therapeutic target at the intersection of metabolic dysregulation, neuroinflammation, and AD pathology.

The autophagy‐lysosome pathway (ALP) is a crucial cellular system responsible for the degradation and recycling of damaged cellular components, including toxic protein aggregates, dysfunctional organelles, and intracellular pathogens. Transcription factors EB (TFEB) and TFE3 are recognized as primary regulators of ALP, with evidence linking deficient TFEB/TFE3‐mediated autophagic processes to AD pathogenesis [16]. Previous studies have underscored the therapeutic potential of TFEB activators, which enhance autophagy and lysosomal biogenesis, reduce toxic protein burden, and alleviate HFD‐induced metabolic syndrome in murine models [17, 18]. Additionally, ALP and the NLRP3 inflammasome appear to interact, wherein ALP can suppress NLRP3 activation through the autophagic degradation of inflammasome components and cytokines [19]. This interrelationship suggests that modulating ALP activity may represent a novel therapeutic avenue to restrict NLRP3 inflammasome activation in both metabolic disorders and AD.

Complementary therapeutic approaches, including acupuncture and electroacupuncture (EA), have shown promise in mitigating symptoms of neurodegenerative diseases. EA at the ST36 acupoint is widely utilized to modulate systemic inflammation, oxidative stress, hormone secretion, and immune function, with emerging applications in AD treatment [20, 21, 22]. Prior work by our group has demonstrated the neuroprotective effects of EA at ST36 (EA‐ST36) in the 3xTg‐AD mouse model [23]. However, the mechanisms by which EA‐ST36 may mitigate metabolic and AD‐related pathologies in HFD‐fed 3xTg‐AD mice remain incompletely understood. This study seeks to elucidate whether EA at ST36 can attenuate HFD‐induced AD pathologies, with a particular focus on the underlying molecular mechanisms involving TFEB/TFE3‐mediated ALP modulation and its relevance to AD and metabolic dysfunction.

## 2 Materials and Methods

### 2.1 Animals

Female triple‐transgenic AD (3xTg‐AD) mice (Stock No. 033930; Jackson Laboratory), co‐expressing human MAPT, APP, and PSEN‐1 transgenes [24], were obtained from the Shenzhen Center for Disease Control and Prevention and housed under standardized conditions (12‐h light/dark cycle, ad libitum access to food and water). At 7 months of age—corresponding to the early pre‐symptomatic phase of AD pathology [23]—mice were randomly assigned to either a normal diet (ND: 18% kcal from fat, 3.1 kcal/g; Teklad) or a high‐fat diet (HFD: 60% kcal from fat [31% lard, 3.7% soybean oil], 20% kcal from protein, 20% from carbohydrates; Research Diets, D12492) for 16 weeks. This HFD duration was selected based on prior studies demonstrating its efficacy in inducing metabolic dysfunction and cognitive deficits in 3xTg‐AD models [8, 25, 26, 27, 28, 29]. Female mice (*n* = 10–12 per group) were exclusively used to ensure phenotypic consistency and to account for their heightened amyloid pathology compared to males [30]. Age‐matched female C57BL/6J mice served as the wild‐type control (WT). All procedures complied with ARRIVE guidelines and were approved by the Animal Care and Use Committee of Guangzhou University of Traditional Chinese Medicine (Approval No. 20230526004).

### 2.2 Electroacupuncture (EA‐ST36) Treatment

Throughout the 4‐month HFD regimen, mice were treated with EA. Behavioral and metabolic assessments were conducted concurrently. At 7 months of age, mice were randomly allocated to either the Sham or EA group. EA stimulation was performed at the ST36 acupoints, following previously established protocols [23]. The parameters (2 Hz frequency, 1 mA intensity, 20 min/session, 5 sessions/week) were selected based on rodent studies demonstrating neuroprotection and anti‐inflammatory effects [31]. The ST36 acupoints, located 5 mm below the fibular head and 2 mm lateral to the anterior tubercle of the tibia, were prepared by cleansing with alcohol, and needles were inserted to a 10 mm depth. Mice were anesthetized with 2% isoflurane (RWD Life Science, R510‐22) and positioned in a stereotaxic apparatus (RWD Life Science, 71,000). An electrical stimulator (Huatuo SDZ, China) was connected to the needle handles, applying the aforementioned parameters over 16 weeks. Sham‐EA animals received superficial needle insertion at ST36 without electrical stimulation. Mice were used for further analyses at 11.5 months of age, after treatment completion.

### 2.3 AAV‐Mediated Tfeb and Tfe3 Knockdown in the Hippocampus

AAV‐mediated hippocampal knockdown of TFEB and TFE3 was performed using shRNA constructs (BrainVTA Co. Ltd., China) to investigate their role in EA‐ST36‐mediated autophagy regulation. The *Tfeb* shRNA sequence (CCGGCGGCAGTACTATGA CTATGATCTCGAG‐ATCATAGTCATAGTACTG CCGTTTTTG) and *Tfe3* shRNA sequence (CCGGGCCTAACATCAAACGCGAGATCTCGAGATCTCGCGTTTGATGTTAGGCTTTTTG) were synthesized based on prior studies [30]. Viral constructs—rAAV‐U6‐shRNA(TFE3)‐U6‐shRNA(TFEB)‐CMV‐EGFP‐SV40 pA, AAV9 (5.87E+12 vg/mL), and control rAAV‐U6‐shRNA(Scramble)‐U6‐shRNA(Scramble)‐CMV‐EGFP‐SV40 pA, AAV9 (5.10E+12 vg/mL)—were produced by BrainVTA Co. Ltd. (Wuhan, China). Female 3xTg‐AD mice (7–8 months, *n* = 36) received bilateral hippocampal CA1 injections (coordinates: −2.06 mm AP, ±1.50 mm ML, 1.4 mm DV from bregma) of 2 μL viral solution at 4 nL/s. Injections were administered two weeks prior to EA‐ST36 treatment, followed by behavioral and biochemical analyses.

### 2.4 Metabolic Analysis

Body weight was monitored weekly throughout the study. After 4 months of treatment, insulin sensitivity and glucose tolerance were evaluated via intraperitoneal insulin tolerance tests (ITTs) and glucose tolerance tests (GTTs), respectively (*n* = 10–12 mice/group). To control for hormonal fluctuations, all tests were performed during the diestrus phase of the estrous cycle in female mice. Following a 6‐h fast, mice received intraperitoneal injections of human insulin (1 unit/kg) or glucose (1 g/kg) [32]. Blood glucose levels were measured from saphenous vein samples using a glucometer (Model 710, Yuwell‐Jiangsu Yuyue Medical Equipment & Supply Co. Ltd) at baseline (*t* = 0) and 15, 30, 60, and 120 min postinjection. The area under the curve (AUC) for glucose concentration was calculated as previously described [33].

### 2.5 Behavioral Assessment

The Morris Water Maze (MWM) was employed to assess hippocampus‐dependent spatial memory, following established protocols [34]. Tests were conducted during the animals' active light phase (07:00–19:00), with each mouse's swim trajectory recorded via a video analysis system (Shanghai Jiliang Software Technology Co. Ltd., Shanghai, China). Pool temperature was maintained at 21°C ± 1°C, and an escape platform (4.5 cm diameter) was submerged 0.8–1 cm below the water surface. Mice underwent five days of training, with four 60‐s trials per day (30‐min intervals between trials). Mice unable to find the platform were placed on it for 10 s. On the sixth day, the platform was removed for a 60‐s probe trial to assess retention.

The Y‐maze test evaluated spatial working memory by measuring spontaneous alternation behavior (SAB), a hippocampal‐dependent metric of memory and attention [35]. Mice were permitted to explore the maze for 5 min or until 15 arm entries. SAB was calculated as the number of alternations divided by total alternation opportunities, using the first 15 arm choices for analysis to control for variability.

### 2.6 Immunohistochemistry

Mice were anesthetized and perfused with 70 mL of ice‐cold PBS, followed by 20 mL of 4% paraformaldehyde (PFA) in PBS. Tissues were fixed overnight at 4°C, and frozen brain sections (40 μm) were prepared using a microtome (HM 525, Leica Biosystems). Sections were permeabilized with 0.3% Triton X‐100 (Beyotime Biotech, ST795) and blocked with 5% donkey serum (Jackson ImmunoResearch Inc., 102,974) in PBS, then incubated with primary antibodies against NLRP3, ASC, or SQSTM1/p62 at dilutions of 1:300 or 1:500 overnight at 4°C. After PBS washes, sections were incubated with Alexa Fluor‐conjugated secondary antibodies (1:1500, Thermo Fisher Scientific, A11008, A11012, A11001, A11005) for 1.5 h at room temperature. Nuclear staining was performed with DAPI, and sections were mounted and imaged using a Nikon A1R confocal microscope (Nikon Instruments Inc.). Images were processed with Adobe Photoshop CS (San Jose, CA, USA) and quantified using ImageJ (NIH, USA).

### 2.7 Tissue Extraction and Western Blot Analysis

The hippocampus was dissected and homogenized in RIPA buffer (1% Triton X‐100, 1% sodium deoxycholate, 0.1% SDS) containing protease and phosphatase inhibitors (Thermo Fisher Scientific, A32957, A32955). Sarkosyl fractionation was conducted to separate soluble and insoluble protein fractions, following previously described protocols [36]. Protein concentrations were determined via BCA assay (Beyotime Biotechnology, P0009). Equal protein amounts were subjected to 8%–15% SDS‐PAGE and transferred onto PVDF membranes (GE Amersham, 10600023). Membranes were blocked with 5% nonfat milk and incubated with primary antibodies overnight, followed by incubation with secondary antibodies for 2 h at room temperature. Signals were detected using ECL kits (Thermo Fisher Scientific, 32106, 34580) and analyzed with ImageJ software.

Antibodies used: Anti‐LAMP1 (ab24170), anti‐CTSD (ab75852), anti‐p‐RELA/ NF‐κB‐p65 (S536) (ab86299), anti‐CASP1 (ab179515), anti‐IL1B (ab9722) and anti‐SQSTM1 (ab109012, used for IHC) were purchased from Abcam.Anti‐NLRP3 (NBP2‐12446), Anti‐LC3B (NB100‐2220) was purchased from Novus Biologicals. Anti‐TFEB (A303‐673A) was purchased from Bethyl Laboratories. Anti‐phospho‐TFEB (Ser142; ABE1971), Anti‐TFE3 (HPA023881) and Anti‐SQSTM1/p62 (P0067, used for WB) were purchased from Sigma‐Aldrich. Anti‐H3F3A/histone H3 (D1H2; 4499), anti‐PSEN1 (5643), anti‐phospho‐MTOR (Ser2448; 2971), anti‐MTOR (2983), anti‐phospho‐AMPKα (Thr172; 2535), anti‐AMPKα (5831), anti‐ULK1(#8054), anti‐phospho‐ULK1 (Ser757) (#6888) were purchased from Cell Signaling Technology. Anti‐AT8 (Ser202/Thr205) (MN1020), anti‐AT270 (Thr181) (MN1050), anti‐AT100(Thr212, Ser214)(MN1060), Anti‐Tau Monoclonal Antibody (HT7)(MN1000), Anti‐APP (51–2700)were purchased from Thermo Fisher Scientific.Anti‐β‐amyloid 1–42 (AB5078P) was purchased from Millipore. Anti‐β‐amyloid (6E10; 803,017) was purchased from Biolegend. Anti‐TUBB/β‐tubulin (H‐235; sc‐9104), anti‐GAPDH (G‐9; sc‐ 365,062), anti‐ASC (sc‐271054), and anti‐ACTB/β‐actin (sc‐47,778) were purchased from Santa Cruz Biotechnology.HRP‐conjugated goat anti‐mouse (115‐035‐003) and goat anti‐rabbit (111–035‐003) secondary antibodies were purchased from Jackson ImmunoResearch.

### 2.8 Statistical Analysis

Data are presented as mean ± SEM. Analyses were conducted using GraphPad Prism 8.0.1. Behavioral data were evaluated using two‐way ANOVA with Bonferroni *post hoc* tests. Comparisons between two groups were conducted using unpaired *t*‐tests, while one‐way ANOVA with Dunnett's multiple comparison test was applied for three or more groups. Outliers were identified via the ROUT method (*Q* = 1%). Significance was set at *p* < 0.05, 0.01, or 0.001.

## 3 Results

### 3.1 EA‐ST36 Attenuates HFD‐Induced Metabolic Dysfunction in 3xTg Mice

Mice were fed either a ND or an HFD for 16 weeks. HFD‐fed 3xTg‐AD mice exhibited significant weight gain compared to the normal diet (ND) group (Figure S1A), consistent with HFD‐induced obesity. EA‐ST36 treatment partially attenuated this weight gain, though the effect was not statistically significant. Metabolic analyses (Figure S1B,C) demonstrated that EA‐ST36 improved glucose tolerance in HFD‐fed mice, suggesting a beneficial effect on metabolic dysfunction. However, insulin sensitivity (Figure S1E) was not significantly altered by HFD or EA‐ST36. These results indicate that EA‐ST36 primarily targets neuropathology rather than systemic metabolism, though metabolic improvements may contribute indirectly to cognitive rescue.

### 3.2 EA‐ST36 Attenuates HFD‐Induced Learning and Memory Deficits in 3xTg‐AD Mice

**FIGURE 1:**
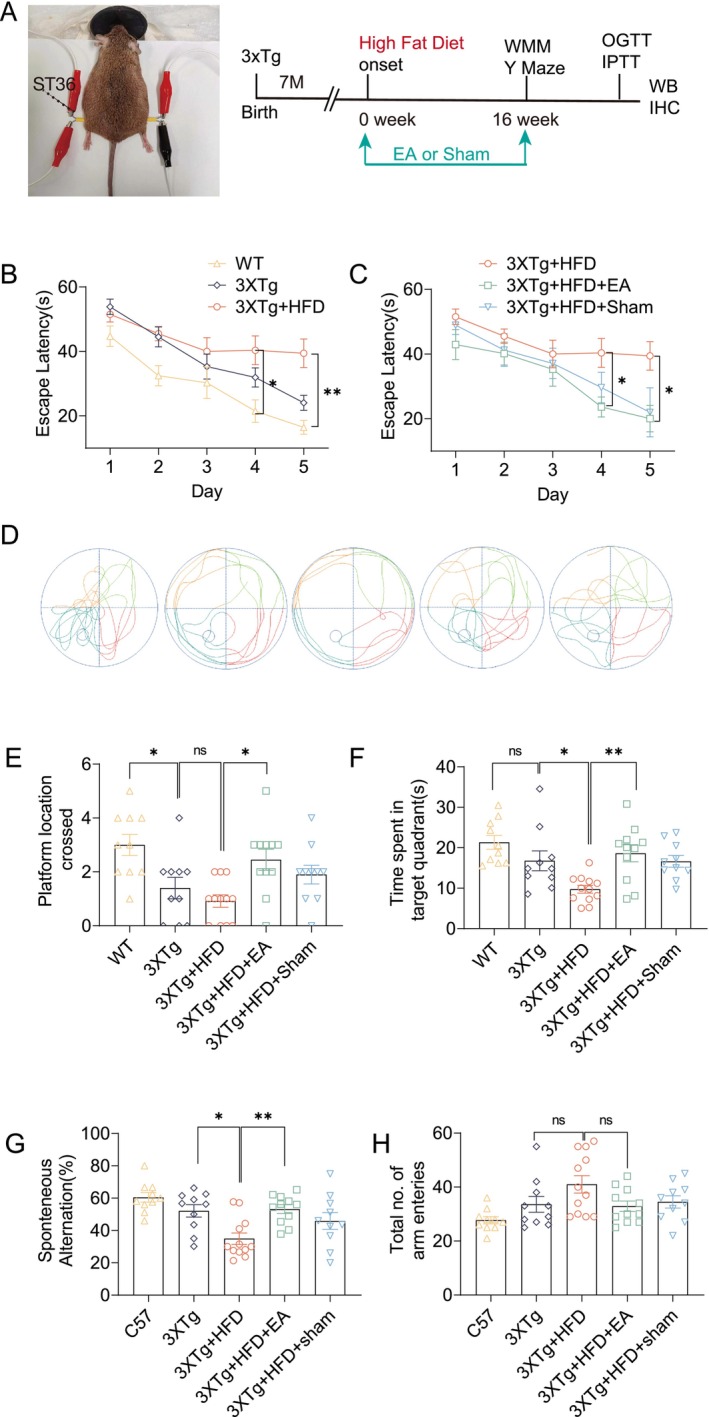
EA‐ST36 alleviates HFD‐induced cognitive dysfunction in 3xTg‐AD mice. (A) Diagram of the EA procedure at acupoint ST36, showing needle placement and electrical stimulation setup. Abbreviations: EA, electroacupuncture; MWM, Morris water maze; WB, western blotting; IHC, immunohistochemistry. (B, C) Morris water maze performance, showing escape latency quantification (mean ± SEM) across groups (males, *n* = 10–12). *p < 0.05, **p < 0.01 versus 3xTg + HFD; analyzed by 2‐way ANOVA with Bonferroni correction. (D) Representative movement paths of each group in the Morris water maze. (E) Quantification of time spent in the target quadrant (mean ± SEM, females, *n* = 10–12) during the probe trial. (F) Quantification of platform crossings (mean ± SEM, females, *n* = 10–12) during the probe trial. *p < 0.05, **p < 0.01 versus. 3xTg + HFD; one‐way ANOVA. (G, H) Y‐maze spontaneous alternation behavior (SAB) in HFD‐fed female 3xTg‐AD mice, with percentage of alternation quantified (mean ± SEM, females, *n* =10‐12 ). *p < 0.05, **p < 0.01 versus 3xTg + HFD; one‐way ANOVA.

To enhance memory deficits, 3xTg‐AD mice were administered a high‐fat diet (HFD) starting at 7 months, a regimen known to exacerbate AD‐like pathology [2]. As anticipated, HFD worsened behavioral disorders and cognitive deficits in these mice. To evaluate the protective effects of EA‐ST36, Morris Water Maze (MWM) and Y‐maze tests were conducted. In the MWM test, HFD‐fed 3xTg‐AD mice exhibited significantly longer escape latencies than mice on a standard diet, particularly on training days 4 and 5 (Figure 1D). Notably, treatment with EA‐ST36 significantly reduced escape latencies on these days, indicating a protective effect (Figure 1D). However, Sham‐EA treatment did not produce a notable reduction in escape latency (Figure 1D). In the probe trial, mice treated with EA‐ST36 spent more time in the target quadrant and crossed the platform area more frequently than those treated with Sham‐EA (Figure 1C,E,F). Spatial working memory was assessed through the Y‐maze task. HFD‐fed 3xTg‐AD mice showed a lower spontaneous alternation percentage compared to mice on a normal diet, suggesting HFD‐induced memory impairment. Importantly, EA‐ST36 treatment significantly restored spatial working memory in HFD‐fed 3xTg‐AD mice (Figure 1G), while Sham‐EA had no effect. No significant differences were observed in the number of arm choices between mice on standard and high‐fat diets (Figure 1H). These findings suggest that EA‐ST36 can ameliorate HFD‐induced deficits in spatial learning and working memory in 3xTg‐AD mice.

### 3.3 HFD Exacerbates Tau Accumulation and NLRP3 Inflammasome Activation in 3xTg‐AD Mice

**FIGURE 2:**
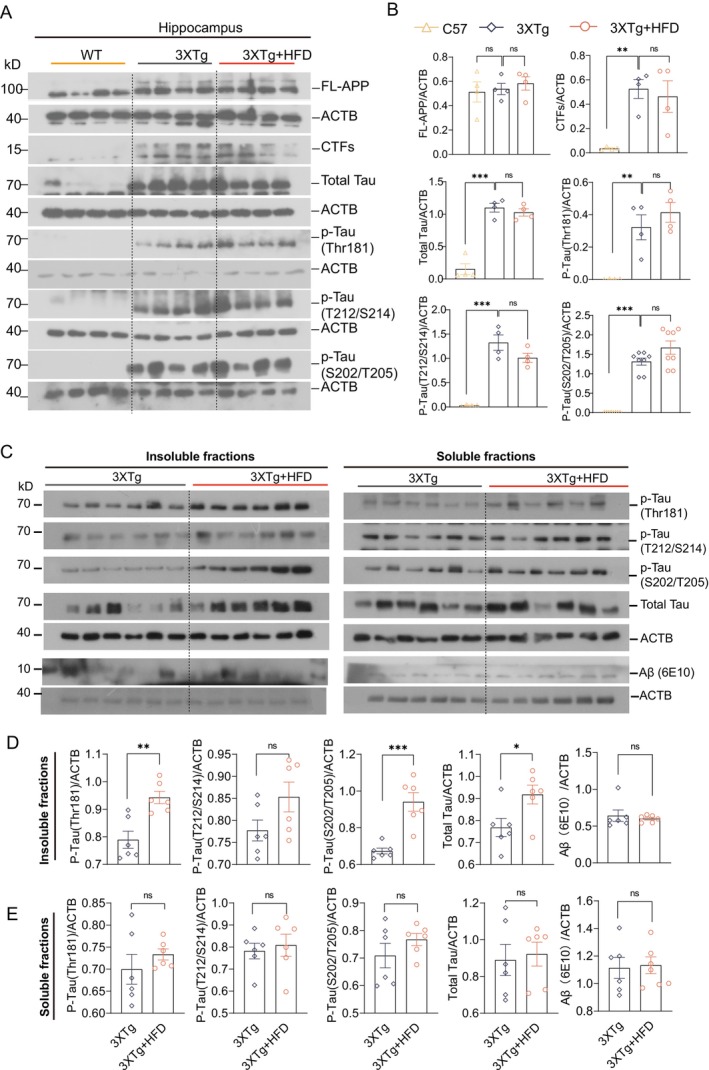
Increase in insoluble Tau aggregates following HFD in female 3xTg‐AD mice. (A) Representative western blots of hippocampal (HI) proteins, showing levels of full‐length APP (FL‐APP), CTF‐β/α, total tau, and phosphorylated tau at Thr181 (AT270), Ser202/Thr205 (AT8), and T212/S214. (B) Quantified data are presented as mean ± SEM. *p < 0.05, **p < 0.01, ***p < 0.001 versus 3xTg (female, *n* = 4), analyzed by one‐way ANOVA. (C) Western blot analysis of Tau and Aβ (6E10) in brain sarkosyl‐insoluble and sarkosyl‐soluble fractions. (D, E) Quantification of Tau and Aβ levels in sarkosyl‐insoluble (D) and sarkosyl‐soluble (E) fractions (mean ± SEM, females, *n* = 6). *p < 0.05, **p < 0.01, ***p < 0.001 versus 3xTg; Unpaired *t*‐test.

Given that 3xTg‐AD mice exhibit both tau and Aβ accumulation in the brain, we explored the mechanisms underlying the exacerbation of the AD phenotype due to HFD by quantifying total tau, phosphorylated tau at Thr181 (AT270), T212/S214, full‐length APP (Fl‐APP), and C‐terminal fragments (CTF‐α/β). As expected, 3xTg‐AD mice showed elevated levels of total and phosphorylated tau in the hippocampus compared to Non‐Tg mice (Figure 2A,B). Similarly, Fl‐APP and CTF levels were higher in 3xTg‐AD mice than Non‐Tg controls (Figure 2A,B). Interestingly, 4 months of HFD exposure did not significantly affect APP/CTF or tau protein levels in 11‐month‐old mice, consistent with previous studies [6, 27, 28]. To further examine tau pathology, we quantified Aβ and tau phosphorylation levels in the soluble and insoluble fractions of hippocampal tissue. As previously reported [5, 29, 37, 38], HFD exposure significantly increased total insoluble tau and its phosphorylation at AT270 and AT8 in the hippocampus of 3xTg‐AD mice (Figure 2C,D). However, HFD exposure did not alter soluble or insoluble Aβ levels (6E10) (Figure 2C–E).

**FIGURE 3:**
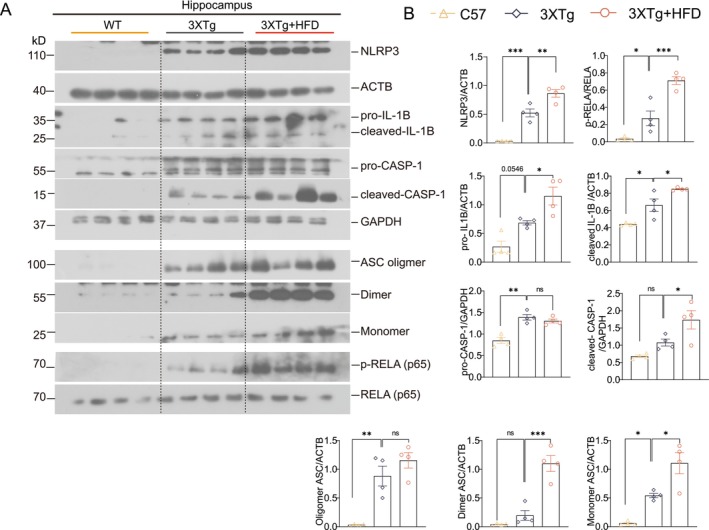
HFD activates the NLRP3 inflammasome in the hippocampus (HI) of 3xTg‐AD mice. (A) Representative western blots of hippocampal proteins, showing levels of NLRP3, IL‐1β, caspase‐1 (CASP1), ASC, phosphorylated RELA (*p*‐RELA), and RELA. (B) Quantified data are presented as mean ± SEM (females, *n* = 6). *p < 0.05, **p < 0.01, ***p < 0.001 versus 3xTg + HFD; one‐way ANOVA.

Prior studies have shown NLRP3 inflammasome activation and elevated IL‐1β levels in AD patients and AD transgenic mouse models, such as Tau P301S (PS19) and 3xTg‐AD mice [39, 40]. While HFD is widely known to worsen brain Aβ and tau pathology, the role of the NLRP3 inflammasome has been less explored. We investigated the effects of HFD on NLRP3 inflammasome activation, observing that HFD exposure activated NF‐κB transcription, resulting in increased levels of NLRP3, pro‐IL‐1β, and ASC. This activation was confirmed by elevated phosphorylated NF‐κB‐p65 (RELA), NLRP3, pro‐/cleaved‐CASP1, and cleaved IL‐1β levels in the hippocampus of HFD‐fed 3xTg‐AD mice (Figure 3A,B). Additionally, ASC oligomers and monomers were elevated, indicating NLRP3–ASC complex formation in the hippocampus of HFD‐fed 3xTg‐AD mice. Together, these findings suggest that HFD‐induced cognitive impairments in 3xTg‐AD mice are closely associated with increased insoluble tau aggregates and NLRP3 inflammasome activation in the hippocampus.

### 3.4 EA‐ST36 Inhibits Tau Aggregation and NLRP3 Inflammasome Formation in HFD‐Exacerbated 3xTg‐AD Mice

**FIGURE 4:**
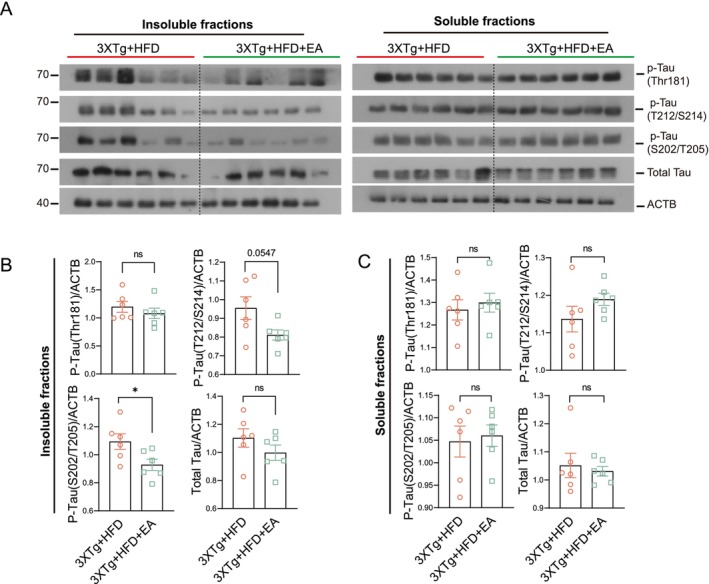
EA‐ST36 promotes degradation of insoluble Tau aggregates in the hippocampus of HFD‐fed 3xTg‐AD mice. (A) Western blots showing levels of total tau and phosphorylated tau at Thr181 (AT270), Ser202/Thr205 (AT8), and T212/S214 in the hippocampus. Lysates were fractionated into sarkosyl‐insoluble and sarkosyl‐soluble components. (B, C) Quantification of tau in sarkosyl‐insoluble (B) and sarkosyl‐soluble (C) fractions (mean ± SEM, females, *n* = 6). *p < 0.05 versus 3xTg + HFD; Unpaired *t*‐test.

To investigate the therapeutic effects of EA‐ST36 on HFD‐aggravated Alzheimer's pathology, we first assessed its impact on tau aggregation. While EA‐ST36 had no significant effect on soluble phosphorylated tau levels in HFD‐fed 3xTg‐AD mice (Figure 4A,B), it markedly reduced sarkosyl‐insoluble phosphorylated tau (AT8) aggregates, indicating enhanced clearance of pathological tau species (Figure 4B).

**FIGURE 5:**
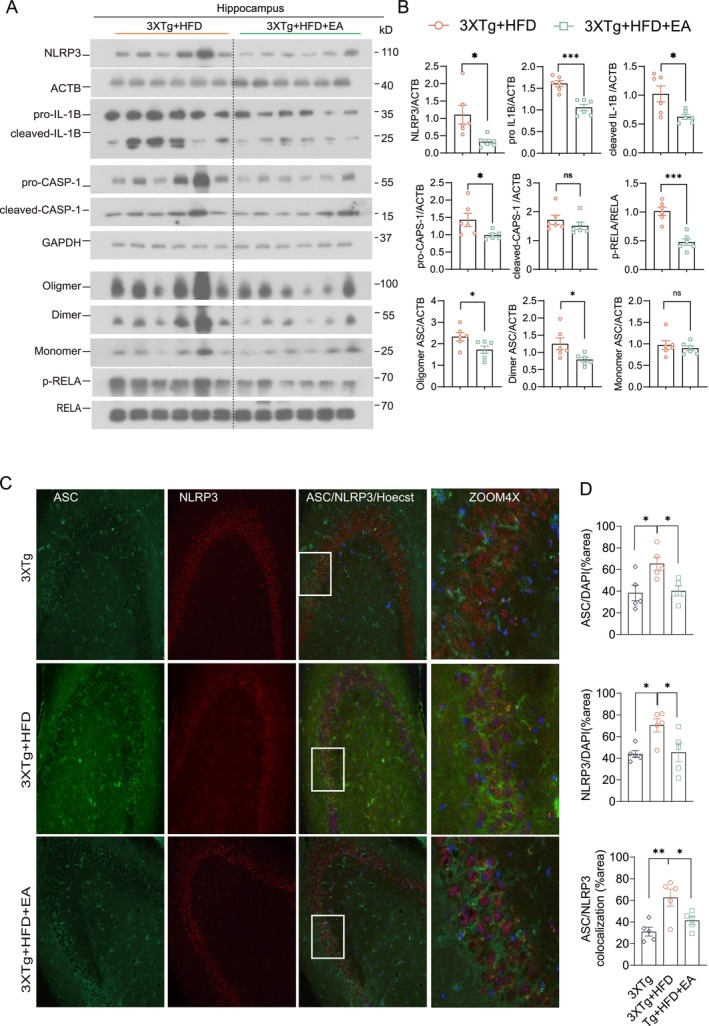
HFD impairs lysosomal function and autophagic flux in the hippocampus of 3xTg‐AD mice. (A) Representative western blots of hippocampal proteins, showing levels of SQSTM1, LAMP1, CTSD, TFEB, TFE3, phosphorylated TFEB (*p*‐TFEB S142), and LC3B, with loading controls. (B) Quantified data are presented as mean ± SEM (*n* = 6). *p < 0.05, **p < 0.01, ***p < 0.001 versus 3xTg + HFD; Unpaired *t*‐test. (C) Representative immunohistochemistry (IHC) images of NLRP3 (red), ASC (green), and nuclei (Hoechst, blue) with colocalization in the hippocampal CA3 region. Scale bar: 100 μm. Enlarged 4× images are shown for the selected area. (D) ImageJ was used to quantify the average areas of NLRP3, ASC, and their colocalization normalized by the area of Hoechst. Data are shown as mean ± SEM (females, *n* = 5); analyzed by one‐way ANOVA. *p < 0.05, **p < 0.01 vs. 3xTg+HFD.

Next, we evaluated EA‐ST36's role in modulating NLRP3 inflammasome activation. Hippocampal levels of NLRP3, pro‐caspase‐1 (pro‐CASP1), and pro‐/cleaved IL‐1β were significantly reduced in EA‐ST36‐treated mice compared to HFD controls (Figure 5A,B). Furthermore, EA‐ST36 diminished ASC dimer/oligomer formation, reflecting suppression of NLRP3–ASC inflammasome assembly (Figure 5B). Sham‐EA failed to reduce FL‐APP, insoluble tau (AT8), or NLRP3–ASC complexes, confirming the specificity of EA‐ST36's effects (Figure S2A–D).

Immunohistochemical analysis further validated these findings, demonstrating reduced NLRP3–ASC colocalization in the hippocampal CA3 region following EA‐ST36 treatment (Figure 5C,D). Additionally, EA‐ST36 decreased phosphorylated NF‐κB‐p65 (RELA) levels, suggesting its inhibitory effects on NLRP3 inflammasome activation may be mediated through the NF‐κB signaling pathway.

### 3.5 EA‐ST36 Promotes Autophagy‐Lysosomal Pathway via TFEB/TFE3 Activation

**FIGURE 6:**
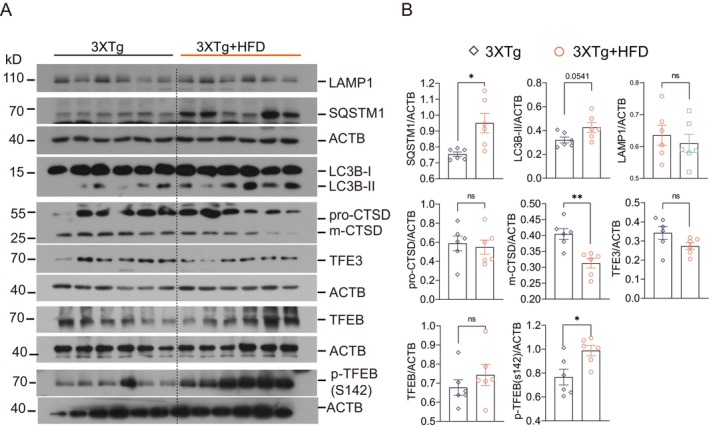
EA‐ST36 promotes degradation of the NLRP3–ASC complex in the hippocampus (HI) of HFD‐fed 3xTg‐AD mice. (A) Representative western blots displaying levels of NLRP3, IL‐1β, caspase‐1 (CASP1), ASC, phosphorylated RELA (*p*‐RELA), and RELA in the hippocampus across groups. (B) Quantified data are presented as mean ± SEM (females, *n* = 6). *p < 0.05, **p < 0.01, ***p < 0.001 versus 3xTg + HFD; Unpaired *t*‐test. (C) Representative immunohistochemistry (IHC) images of NLRP3 (red), ASC (green), and nuclei (Hoechst, blue) with colocalization in the hippocampal CA3 region. Scale bar: 100 μm. Enlarged 4× images are shown for the selected area. (D) ImageJ was used to quantify the average areas of NLRP3, ASC, and their colocalization normalized by the area of Hoechst. Data are shown as mean ± SEM (females, *n* = 5); analyzed by one‐way ANOVA. *p < 0.05, **p < 0.01 versus 3xTg + HFD.

We then investigated the mechanisms by which HFD exacerbates AD pathology in 3xTg‐AD mice. Impaired autophagic flux, characterized by sustained autophagy induction but defective lysosomal clearance, has been observed in HFD‐induced nonalcoholic fatty liver disease [41] and brain tissues from 3xTg‐AD mice [42]. Additionally, tau aggregates and NLRP3 inflammasome components are known to degrade through TFEB/TFE3‐mediated autophagy‐lysosome pathways (ALP) [39]. In this study, we explored lysosomal dysfunction and impaired autophagic flux in HFD‐fed 3xTg‐AD mice by assessing total and phosphorylated TFEB/TFE3 levels and ALP targets, including MAP1LC3B/LC3B, SQSTM1/p62, CTSD (cathepsin D), and LAMP1 in hippocampal lysates. After 4 months of HFD exposure, 3xTg‐AD mice displayed significantly increased SQSTM1/p62 and phosphorylated TFEB (p‐TFEB S142), with a trend towards elevated LC3B‐II expression that did not reach statistical significance (Figure 6A,B). Furthermore, these mice exhibited a marked reduction in mature CTSD (m‐CTSD), suggesting inhibition of late‐stage autophagy, resulting in autophagosome accumulation and increased LC3‐II expression on autophagosome membranes. These findings indicate that the worsening of AD pathology in HFD‐fed 3xTg‐AD mice may be attributed to deficient autophagic degradation.

**FIGURE 7:**
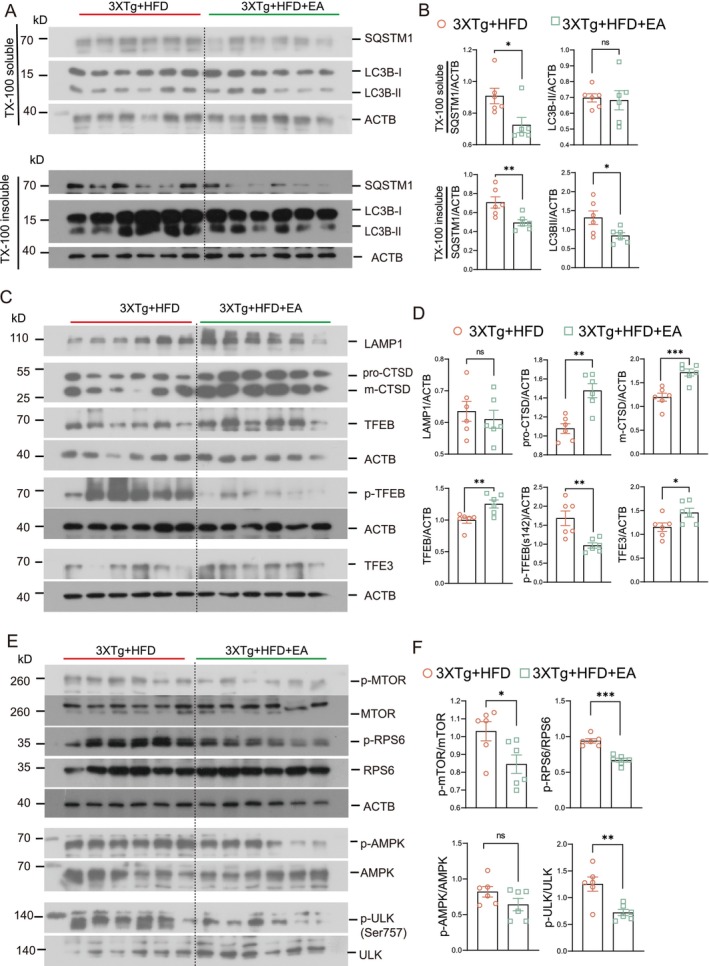
EA‐ST36 increases TFEB/TFE3 expression and activates the autophagy‐lysosomal pathway in the hippocampus (HI) of HFD‐fed 3xTg‐AD mice. (A) Representative western blots of autophagy markers (LC3B and SQSTM1) in TX‐100 soluble and insoluble fractions from the hippocampus. (B, D) Quantified data are shown as mean ± SEM (females, *n* = 6). *p < 0.05 versus 3xTg + HFD; Upaired *t*‐test. (C) Western blots showing levels of TFEB, TFE3, and lysosome markers (LAMP1 and CTSD) in the prefrontal cortex (PFC) and hippocampus. (D) Data quantified as mean ± SEM (females, *n* = 6). *p < 0.05, **p < 0.01, ***p < 0.001 versus 3xTg + HFD; Unpaired *t*‐test. (E‐F) EA‐ST36 treatment inhibits MTORC1 and ULK signaling in the brains of 3xTg + HFD mice. Western blots and quantification show levels of phosphorylated and total MTOR, RPS6, ULK, and AMPK in the hippocampus. (G) Quantified data as mean ± SEM (females, *n* = 6). *p < 0.05, **p < 0.01, ***p < 0.001 versus 3xTg + HFD; Unpaired *t*‐test.

Given that tau aggregates and inflammasome components (NLRP3, ASC, and pro‐IL‐1β) can be degraded through the ALP, we evaluated whether EA‐ST36 reduces NLRP3 inflammasome components via autophagic degradation. To assess the impact of EA‐ST36 on autophagy markers, we measured LC3B and SQSTM1 levels in Triton X‐100‐soluble and SDS‐soluble hippocampal lysates, following established guidelines [30]. Our findings showed no significant change in LC3B‐II levels in the TX‐100‐soluble fractions between the 3xTg + HFD and 3xTg + HFD + EA‐ST36 groups (Figure 7A,B). However, both LC3B‐II and SQSTM1 were significantly reduced in the TX‐100‐insoluble fractions in the 3xTg + HFD + EA‐ST36 group (Figure 7A,B). Regarding lysosomal markers, EA‐ST36 treatment significantly increased both pro‐ and mature CTSD levels, though no significant changes were observed in LAMP1 levels (Figure 7C,D). These findings suggest that EA‐ST36 promotes lysosomal biogenesis, facilitating degradation of insoluble SQSTM1 and tau aggregates. Further analysis demonstrated that EA‐ST36 increased total TFEB levels and significantly elevated total TFE3 levels in the hippocampus of HFD‐fed 3xTg‐AD mice. Additionally, EA‐ST36 treatment significantly reduced phosphorylation of TFEB at S142, a key site regulated by mTORC1 and AMP [43](Figure 7C,D). Collectively, these data suggest that EA‐ST36 activates TFEB/TFE3 to promote lysosomal biogenesis in HFD‐fed 3xTg‐AD mice.

### 3.6 EA‐ST36 Activates TFEB/TFE3 by Inhibiting mTOR/ULK1 Signaling

To further understand how EA‐ST36 activates TFEB/TFE3 in HFD‐fed 3xTg‐AD mice, we examined upstream kinases, including mTORC1 and AKT. Interestingly, EA‐ST36 significantly reduced phosphorylation of mTOR and its substrate, ribosomal protein S6 (RPS6), along with ULK1 (S757) in the hippocampus of HFD‐fed 3xTg‐AD mice (Figure 7E,F). However, EA‐ST36 had no effect on AKT phosphorylation or AMPK phosphorylation in the hippocampus of HFD‐fed 3xTg‐AD mice (Figure 7E,F). These results suggest that EA‐ST36 activates TFEB/TFE3 by inhibiting the mTOR/ULK1 signaling pathway.

### 3.7 EA‐ST36 Facilitates Autophagic Degradation of ASC Oligomers

**FIGURE 8:**
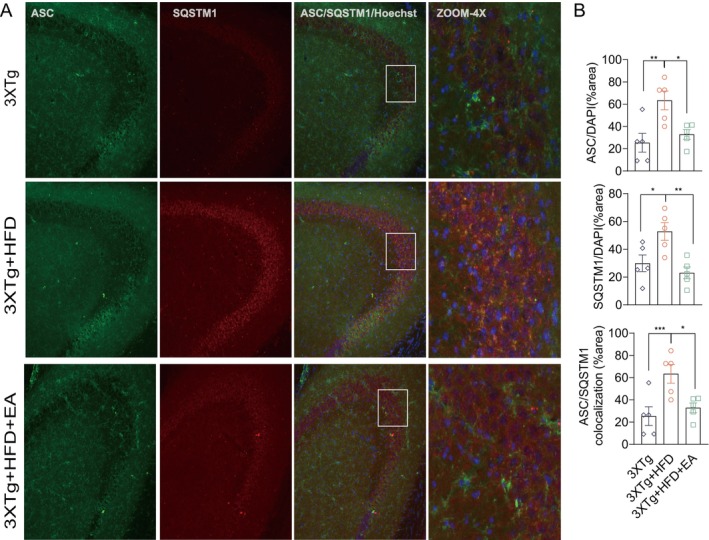
EA‐ST36 promotes autophagic recognition and degradation of ASC specks in the hippocampus (HI) of HFD‐triggered 3xTg‐AD mice. (A) Representative IHC images of ASC (green), SQSTM1/p62 (red), and nuclei (Hoechst, blue) with colocalization in the hippocampal CA3 region. Scale bar: 100 μm. Enlarged 4× images are shown for the selected area. (B) ImageJ quantification of the average areas of SQSTM1, ASC, and their colocalization normalized by the area of Hoechst. Data are presented as mean ± SEM (females, *n* = 5); one‐way ANOVA. *p < 0.05, **p < 0.01 versus 3xTg + HFD.

For autophagic degradation, SQSTM1 binds to LC3 to facilitate degradation of ubiquitinated ASC protein aggregates via autophagy [44]. To determine whether HFD‐induced ASC degradation is associated with ALP, we evaluated ASC and SQSTM1 colocalization in hippocampal regions of 3xTg mice fed with or without HFD. We observed large ASC specks colocalized with SQSTM1 puncta in the hippocampi of HFD‐fed 3xTg mice (Figure 8A,B), indicating accumulation of undigested ASC–SQSTM1 aggregates due to impaired autophagic degradation. Conversely, EA‐ST36 treatment significantly reduced ASC area and ASC‐associated SQSTM1 (Figure 8A,B), indicating that EA‐ST36 promotes autophagic recognition and degradation of ASC oligomers via SQSTM1. These findings suggest that EA‐ST36 facilitates degradation of NLRP3 inflammasome components through the TFEB/TFE3‐mediated autophagy‐lysosomal pathway.

### 3.8 EA‐ST36‐Induced Cognitive Improvements Depend on TFEB/TFE3

**FIGURE 9:**
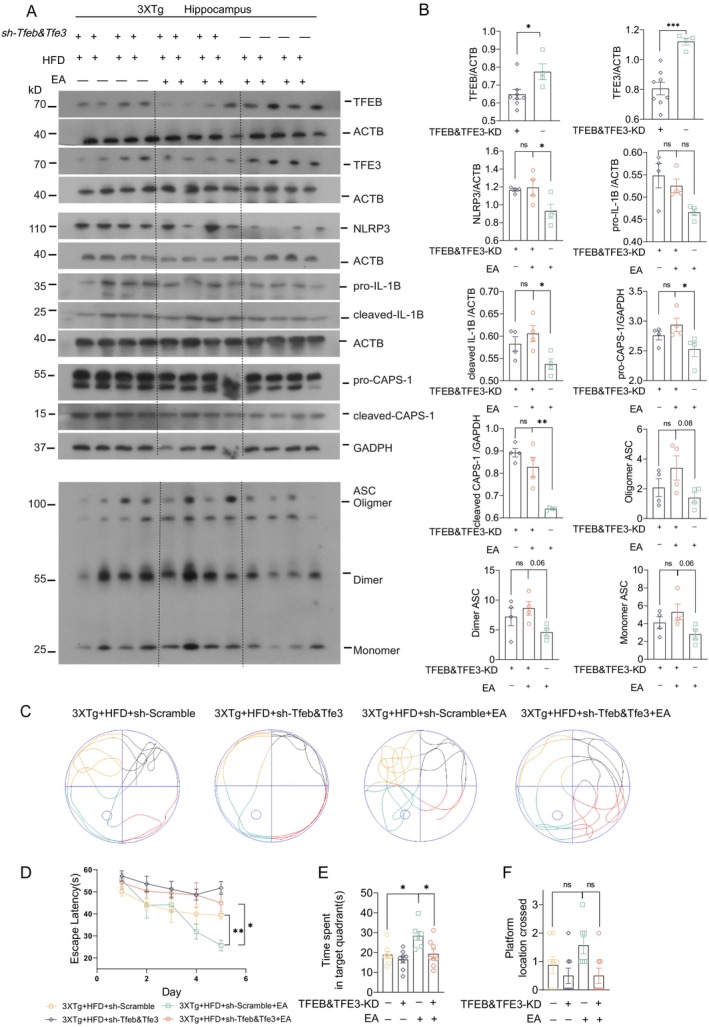
EA‐ST36 enhances autophagic degradation of NLRP3 inflammasome components and improves memory, mediated by TFEB/TFE3. (A) Representative Western blots showing TFEB, TFE3, NLRP3, IL‐1β, CASP1, and ASC levels in the hippocampus of each group, with or without TFEB/TFE3 knockdown (KD). (B) Quantified data are presented as mean ± SEM (females, *n* = 4). *p < 0.05, **p < 0.01, ***p < 0.001, ns (*p* > 0.05) versus EA + TFEB&TFE3‐KD group; analyzed by Unpaired *t*‐test (TFEB&TFE3‐KD+ vs. TFEB&TFE3‐KD−) and one‐way ANOVA. (C–F) Morris water maze assessment. (C) Representative movement patterns for each group. (D) Quantification of escape latencies (mean ± SEM, females, *n* = 7–8). *p < 0.05, **p < 0.01 versus EA + TFEB&TFE3‐KD; analyzed by 2‐way ANOVA with Bonferroni multiple comparison. (E) Quantification of time spent in the target quadrant (mean ± SEM, females, *n* = 7–8) during the probe trial. (F) Quantification of platform crossings (mean ± SEM, females, *n* = 7–8) during the probe trial. *p < 0.05 versus EA + TFEB&TFE3‐KD; analyzed by one‐way ANOVA.

**FIGURE 10:**
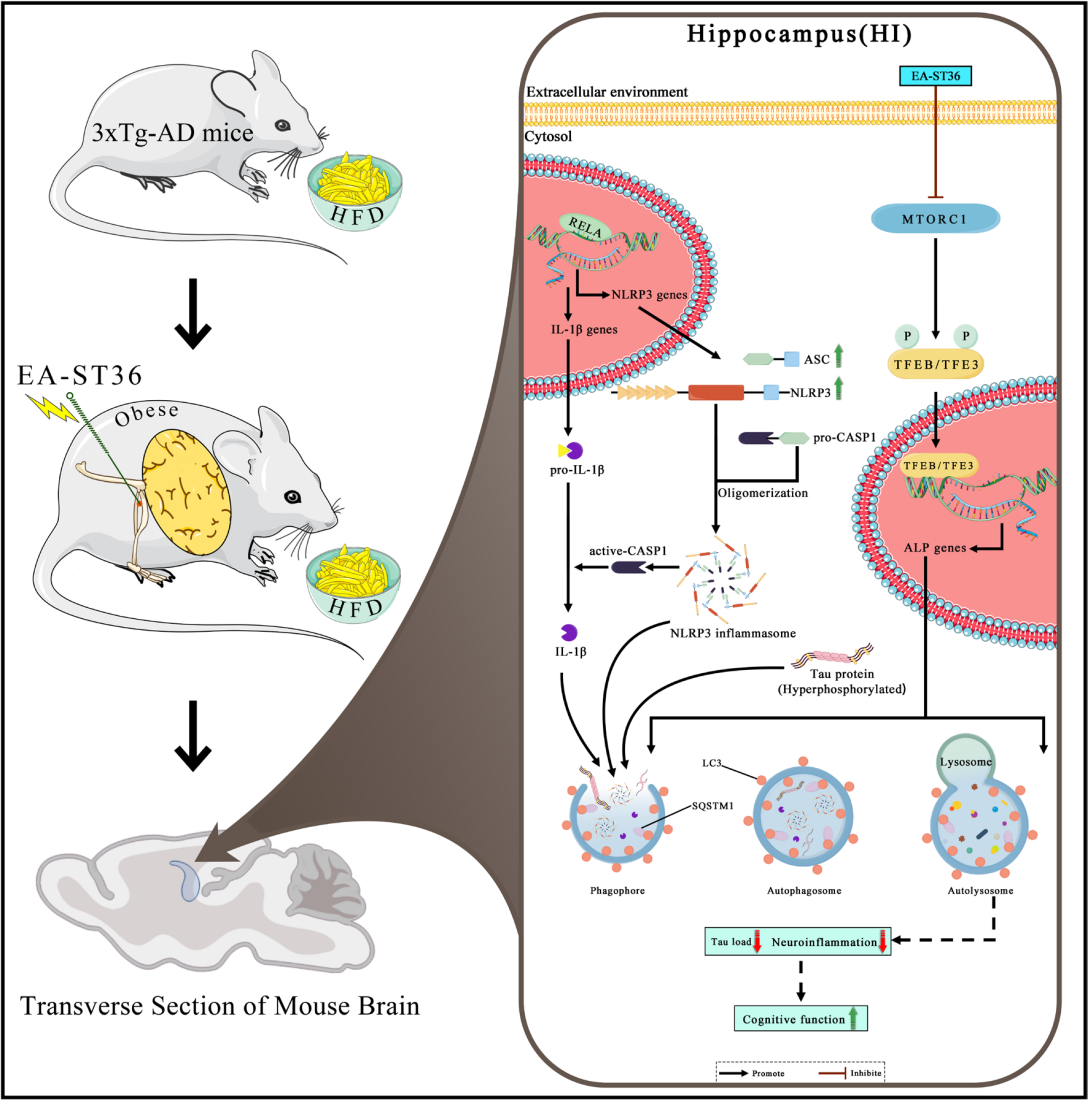
Mechanistic model of EA‐ST36‐mediated reversal of HFD‐induced cognitive impairment in 3xTg‐AD mice through TFEB/TFE3 activation. The model illustrates that EA‐ST36 inhibits MTORC1 and activates TFEB/TFE3, enhancing the autophagy‐lysosomal pathway (ALP), which leads to the effective degradation of Tau aggregates and NLRP3 inflammasome components in the hippocampi of HFD‐fed 3xTg‐AD mice.

Lastly, we determined the specific roles of TFEB/TFE3 in EA‐ST36‐induced NLRP3 inflammasome degradation and memory improvement in HFD‐exacerbated 3xTg‐AD mice. To achieve this, AAV‐sh‐Scramble and AAV‐sh‐Tfeb&Tfe3 were bilaterally injected into hippocampal CA1 regions. Strong GFP signals in the hippocampus confirmed efficient delivery of shRNAs (Figure S1A). However, hippocampal injection of AAV‐sh‐Tfeb&Tfe3, but not AAV‐sh‐Scramble, resulted in high mortality rates (~20%) (Figure S2B). Western blots confirmed TFEB&TFE3 knockdown in surviving mice (Figure 9A,B). In TFEB/TFE3 double‐knockdown (TFEB&TFE3‐KD) mice, EA‐ST36 treatment did not reduce NLRP3, cleaved‐IL1B, or pro‐/cleaved‐CASP1 levels (Figure 9A,B). Notably, the reduction of pro‐IL1B by EA‐ST36 was unaffected by TFEB&TFE3‐KD, indicating that TFEB/TFE3‐mediated ALP is not essential for pro‐IL1B turnover (Figure 9A,B). Importantly, TFEB&TFE3‐KD also abolished EA‐ST36's ability to reduce phosphorylated tau (AT8) in the hippocampus, as shown in Figure S2, confirming that TFEB/TFE3 activation is critical for both NLRP3 inflammasome degradation and tau clearance. Furthermore, the EA‐ST36‐induced improvement in spatial learning memory was blocked in TFEB&TFE3‐KD mice (Figure 9C–F). These results indicate that TFEB/TFE3 are required for EA‐ST36‐induced degradation of NLRP3 inflammasome components and memory improvement. This dual mechanism—reducing neuroinflammation and tauopathy—likely synergizes to mitigate HFD‐induced cognitive deficits (Figure 10).

## 4 Discussion

The dysfunction of the TFEB/TFE3‐mediated autophagy‐lysosomal pathway (ALP) plays a critical role in the pathogenesis of AD and high‐fat diet (HFD)‐induced nonalcoholic fatty liver disease (NAFLD) [45]. The activation of the NLRP3 inflammasome has been identified as a significant factor in obesity‐induced inflammation and insulin resistance [46]. However, few studies have directly examined the relationship between NLRP3‐mediated neuroinflammatory responses and ALP dysfunction in AD pathologies exacerbated by HFD. Recent research demonstrates that TFEB overexpression and small‐molecule TFEB activators can attenuate NLRP3‐driven inflammation in AD animal models, indicating that targeting ALP—particularly through master regulators TFEB and TFE3—may suppress neuroinflammation and mitigate HFD‐induced metabolic and cognitive impairments [47].

In this study, we show that EA‐ST36, a nonpharmacological electroacupuncture therapy, reduces HFD‐induced tau hyperphosphorylation and NLRP3 inflammasome activation in the hippocampus of 3xTg‐AD mice. Notably, this is the first study to demonstrate that EA‐ST36 reverses HFD‐induced AD‐related pathology by promoting the autophagic degradation of inflammasome components (NLRP3 and ASC) and pro‐IL1B through the activation of TFEB and TFE3, mediated by MTORC1 inhibition (Figure 9).

The 3xTg‐AD mouse model is essential in AD pathology research due to the presence of APP, tau, and Psen1 genes associated with familial ad [ 48]. Female 3xTg‐AD mice, in particular, display heightened amyloid pathology, increased beta‐secretase activity, and accelerated cognitive decline compared to males, which makes them especially suitable for AD research [49]. Consistent with previous findings [8, 25, 26, 27, 28, 29], we observed that long‐term HFD consumption impaired learning and memory and induced tau hyperphosphorylation in 3xTg‐AD mice. Although no significant differences in hippocampal Aβ levels were observed between groups, this discrepancy may stem from variations in Aβ detection methods or differences in dietary fat composition. Importantly, EA‐ST36 mitigated HFD‐induced tau hyperphosphorylation and improved memory deficits, as indicated by enhanced performance in the WMM and Y‐maze tests. The absence of statistically significant memory improvement in sham‐EA‐treated mice, coupled with their inability to reduce pathological markers (Figure S2), underscores that EA‐ST36's benefits require active electrical stimulation. This aligns with prior evidence that low‐frequency EA modulates neural circuits and inflammatory pathways distinct from sham interventions [23].

Autophagy dysfunction is increasingly understood as a driver of excessive NLRP3 inflammasome activation, suggesting ALP as a critical regulator of inflammasome dynamics. Autophagic degradation of NLRP3 components and proinflammatory cytokines can attenuate inflammasome activation and inflammatory responses [19]. Our study uniquely demonstrates that HFD concurrently aggravates hippocampal NLRP3 activation and ALP impairment in 3xTg‐AD mice, driving cognitive deficits (Figures 3 and 6). EA‐ST36 mitigated these effects by promoting NLRP3 degradation and reducing insoluble phosphorylated tau aggregates, correlating with improved cognitive performance (Figures 4 and 5). Mechanistically, HFD‐induced metabolic stress synergistically exacerbates tau pathology and NLRP3‐driven neuroinflammation: NLRP3 activation accelerates tau hyperphosphorylation via IL‐1β/NF‐κB signaling [10], while tau aggregates activate glial cells to amplify inflammasome signaling [50, 51]. Although the temporal sequence remains unresolved, this bidirectional interplay underscores HFD's role in bridging metabolic dysfunction and neurodegeneration. Future studies employing time‐course analyses and cell‐specific interventions are needed to delineate causal relationships and refine therapeutic strategies targeting ALP in AD.

Additionally, EA‐ST36 facilitated NLRP3 degradation by activating both TFEB and TFE3 in the hippocampus of HFD‐fed 3xTg‐AD mice. TFEB and TFE3 knockdown (~30%) significantly impeded autophagic degradation of NLRP3 inflammasome components, subsequently hindering memory improvement (Figure 9). However, whether TFEB and TFE3 have equivalent or distinct roles in the autophagic degradation of the NLRP3 inflammasome remains unclear. Previous studies report differential nuclear localization of TFEB and TFE3 in hippocampal neurons and glia, suggesting that TFEB primarily functions in glial cells, while TFE3 functions in neurons [16]. Investigating TFEB and TFE3 nuclear localization in neurons and glia in HFD‐fed 3xTg‐AD mice, with and without EA treatment, will be essential for elucidating their distinct roles in regulating ALP and the NLRP3 inflammasome. Neuron‐ and glia‐specific knockout (KO) models for TFEB and TFE3 should be employed to validate these findings, particularly in relation to the observed reduction in microglial activation in HFD‐fed 3xTg‐AD mouse brains.

EA‐ST36 also promotes autophagy by modulating the mTOR/ULK1 signaling pathway (Figure 7). Prior research has demonstrated that AMPK and mTOR regulate autophagy through the direct phosphorylation of ULK1 [52]. Under nutrient‐rich conditions, elevated mTOR activity inhibits ULK1 activation by phosphorylating ULK1 at Ser757, thus preventing interaction with AMPK. Our results indicate that EA‐ST36‐induced activation of TFEB and TFE3 may be mediated via inhibition of the mTOR/ULK1 pathway. Similar findings have been reported in APP/PS1 mice, where autophagic activation in neurons and microglia alleviated amyloid pathology and cognitive impairment through the AMPK/mTOR/ULK1 pathway [53]. However, the mechanism by which EA‐ST36 avoids AMPK activation remains unclear, highlighting the need for further research into whether EA‐ST36 inhibits neuroinflammation through TFEB‐independent pathways.

In summary, our findings demonstrate that prolonged HFD consumption exacerbates tau phosphorylation and NLRP3 inflammasome activation in the brain, leading to cognitive decline in 3xTg‐AD mice. EA‐ST36 mitigates these harmful effects by activating the TFEB/TFE3‐mediated autophagy‐lysosomal pathway. These results suggest that EA‐ST36 holds promise as a nonpharmacological intervention for preventing cognitive impairment associated with metabolic disorders and may serve as a safe therapeutic approach for delaying AD progression.

## Author Contributions

Xiaoyan Zheng, Zhihao Luo, Jialin Zheng, Chuyu Deng, and Dongmin Liu conducted mice experiments and data acquisition. Zhihao Luo and Chuyu Deng conducted behavioral tests. Dongmin Liu contributed to data collection. Qi Zeng contributed to data analysis and manuscript revision. Ju‐Xian Song, Chunzhi Tang, and Xiaoyan Zheng contributed to the project concept and design, manuscript revision, and funding support. Chunzhi Tang, Ju‐Xian Song, Xiaoyan Zheng, Jihua Zou, and Guozhi Huang contributed to the project concept and design, data interpretation, manuscript writing, and financial support. All authors have read and approved the final manuscript.

## Ethics Statement

All animal care and experimental procedures were approved by the Animals Care and Use Committee of Guangzhou University of Chinese Medicine, in accordance with the ARRIVE guidelines and the Guide for the Care and Use of Laboratory Animals recommended by National Institutes of Health. This article did not contain any studies with human participants.

## Consent

The authors have nothing to report.

## Conflicts of Interest

The authors declare no conflicts of interest.

## Supporting information


**FIGURE S1.** EA‐ST36 promotes glucose tolerance in 3XTg mice fed with high‐fat diet. (A) Body weight gain (g) was measured weekly during 16 weeks in wild type (WT) mice fed with normal diet (ND) or high‐fat diet (HFD) and those that received EA treatment or sham EA treatment: WT fed with a normal diet (WT + ND) or 3XTg mice fed with a normal diet (3xTg + ND) or 3XTg mice fed with a high‐fat diet (3xT + HFD). Data are shown as mean ± SEM, *n* = 10–11. Statistical analysis was performed by two‐way ANOVA with repeated measures followed by *post hoc* Bonferroni’s multiple comparisons test. (B) Blood glucose levels were measured at several time points following insulin administration during the insulin resistance test (IRT) of WT or 3XTg mice 11 months after of being fed with ND or HFD alone or treated with EA. (C) Blood glucose during intraperitoneal glucose tolerance test (GTT) of WT or mice 11 months after of being fed with ND or HFD alone or treated with EA. (D) Bar graph (lower panel) represents the area under the curve (AUC). Data are shown as mean ± SEM, *n* = 10 ~ 11 for all the groups. *p < 0.05 versus 3XTg + HFD group analyzed by one‐way ANOVA followed by Tukey’s multiple comparisons test.
**FIGURE S2.** Sham‐EA does not reduce FL‐APP, tau aggregates, or NLRP3–ASC complexes in HFD‐fed 3xTg‐AD mice. (A) Representative western blots of hippocampal (HI) proteins, showing levels of full‐length APP (FL‐APP), CTF‐β/α, total tau, and phosphorylated tau at Ser202/Thr205 (AT8). (B) Quantified data are presented as mean ± SEM. *p < 0.05 versus 3xTg + HFAD (female, *n* = 6), analyzed by Unpaired *t*‐test. (C) Representative western blots showed the levels of NLRP3, IL1B, caspase‐1 (CASP1) and ASC in the HI of mice from each group. (D) Data are quantified as mean ± SEM (female, *n* = 6). *p < 0.05, ns (*p* > 0.05) versus 3XTg + HFD group analyzed by Unpaired *t*‐test.
**FIGURE S3.** Viral delivery and survival rate assessment postinjection. (A) Representative image showing hippocampal targeting after viral injection. AAV expressing GFP was injected into the bilateral CA1 region of the mouse brain, with green fluorescence indicating viral vector spread throughout the CA1 region. (B) Survival rate quantification of sh‐Scramble and sh‐TFEB&TFE3 groups postinjection. Monitoring began at the injection date and continued until the end of the intervention period. Survival curves demonstrate a difference between sh‐TFEB&TFE3 and sh‐Scramble groups (*p* = 0.0918, Hazard ratio = 3.701 [0.7467, 18.34]).

## Data Availability

All data are available in the main text. Uncropped/unprocessed scans of all immunoblots in the paper are included as Additional file 1, respectively. Other information that supports the findings of this study is available from the corresponding author upon reasonable request.
