# Supplementary figures and images for: Electroacupuncture Attenuates High‐Fat Diet‐Exacerbated Alzheimer's Pathology by Enhancing TFEB/TFE3‐Mediated Autophagic Clearance of Tau and NLRP3 Inflammasome in 3xTg Mice
Source: CNS Neurosci Ther. 2025 Jul 1;31(7):e70497. doi: 10.1111/cns.70497 (PMC12209595; doi:10.1111/cns.70497)

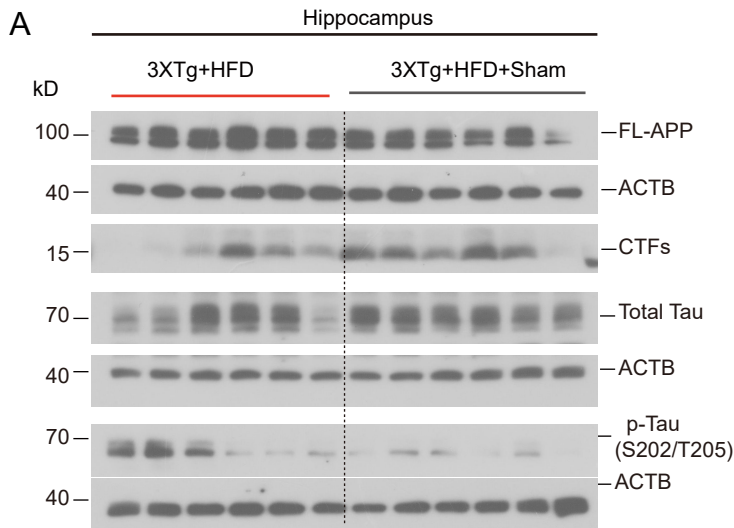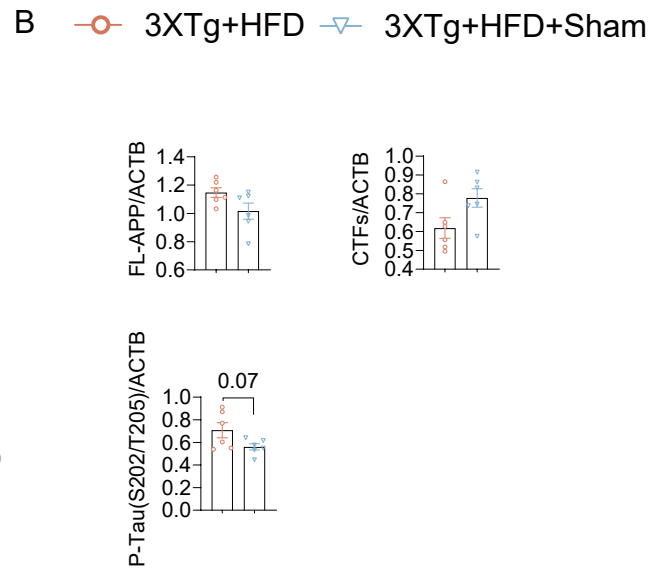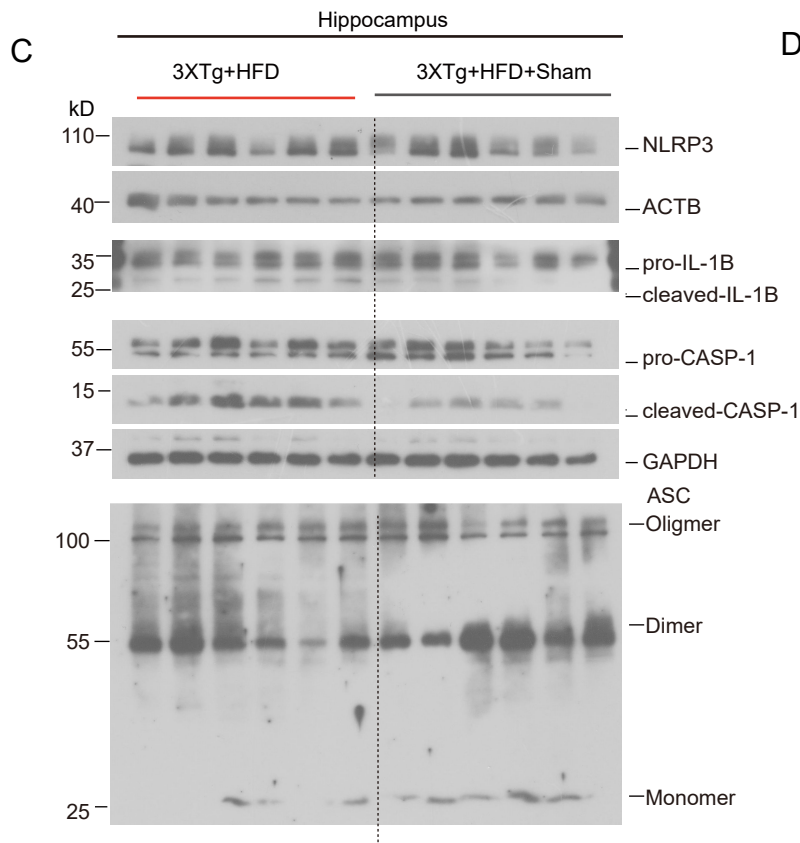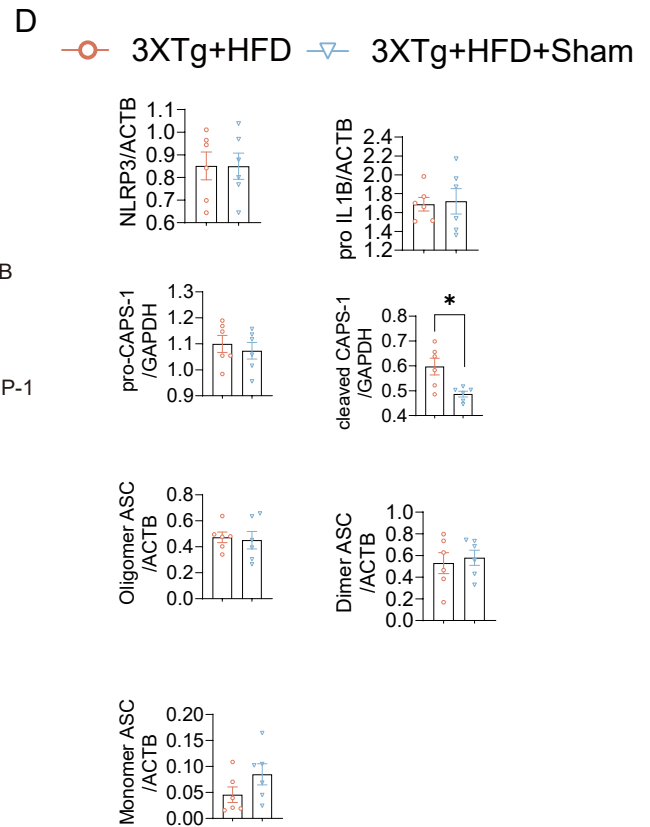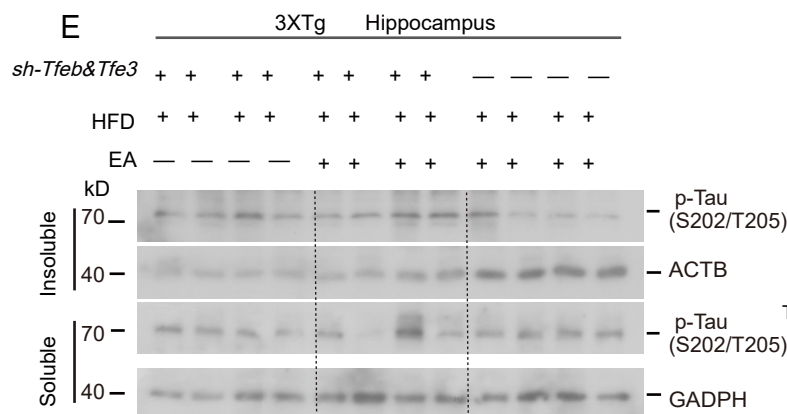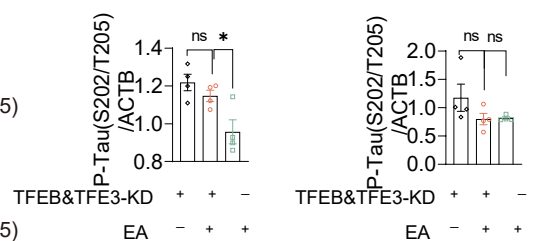

Supplement: Supplementary file 1 — FIGURE S1. EA‐ST36 promotes glucose tolerance in 3XTg mice fed with high‐fat diet. (A) Body weight gain (g) was measured weekly during 16 weeks in wild type (WT) mice fed with normal diet (ND) or high‐fat diet (HFD) and those that received EA treatment or sham EA treatment: WT fed with a normal diet (WT + ND) or 3XTg mice fed with a normal diet (3xTg + ND) or 3XTg mice fed with a high‐fat diet (3xT + HFD). Data are shown as mean ± SEM, n = 10–11. Statistical analysis was performed by two‐way ANOVA with repeated measures followed by post hoc Bonferroni’s multiple comparisons test. (B) Blood glucose levels were measured at several time points following insulin administration during the insulin resistance test (IRT) of WT or 3XTg mice 11 months after of being fed with ND or HFD alone or treated with EA. (C) Blood glucose during intraperitoneal glucose tolerance test (GTT) of WT or mice 11 months after of being fed with ND or HFD alone or treated with EA. (D) Bar graph (lower panel) represents the area under the curve (AUC). Data are shown as mean ± SEM, n = 10 ~ 11 for all the groups. *p < 0.05 versus 3XTg + HFD group analyzed by one‐way ANOVA followed by Tukey’s multiple comparisons test. FIGURE S2. Sham‐EA does not reduce FL‐APP, tau aggregates, or NLRP3–ASC complexes in HFD‐fed 3xTg‐AD mice. (A) Representative western blots of hippocampal (HI) proteins, showing levels of full‐length APP (FL‐APP), CTF‐β/α, total tau, and phosphorylated tau at Ser202/Thr205 (AT8). (B) Quantified data are presented as mean ± SEM. *p < 0.05 versus 3xTg + HFAD (female, n = 6), analyzed by Unpaired t‐test. (C) Representative western blots showed the levels of NLRP3, IL1B, caspase‐1 (CASP1) and ASC in the HI of mice from each group. (D) Data are quantified as mean ± SEM (female, n = 6). *p < 0.05, ns (p > 0.05) versus 3XTg + HFD group analyzed by Unpaired t‐test. FIGURE S3. Viral delivery and survival rate assessment postinjection. (A) Representative image showing hippocampal targeting aft [file CNS-31-e70497-s001.zip › cns70497-sup-0003-FigureS2@FigureS2.pdf]

A

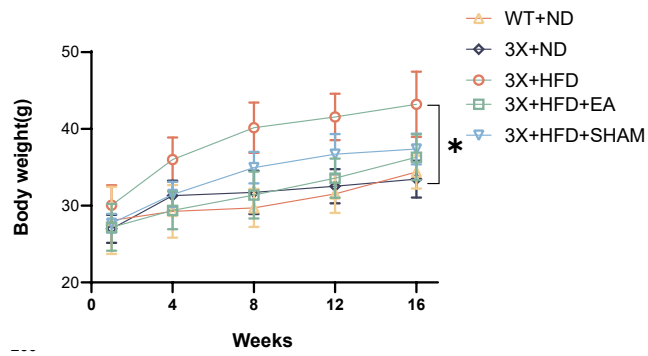

B

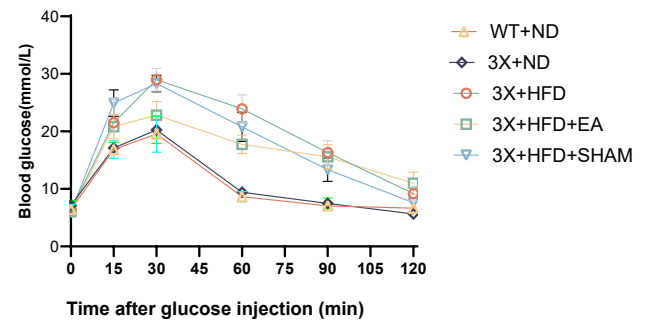

C

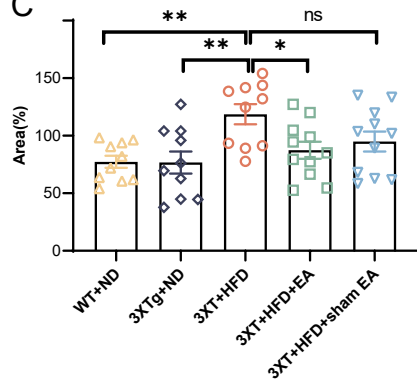

D

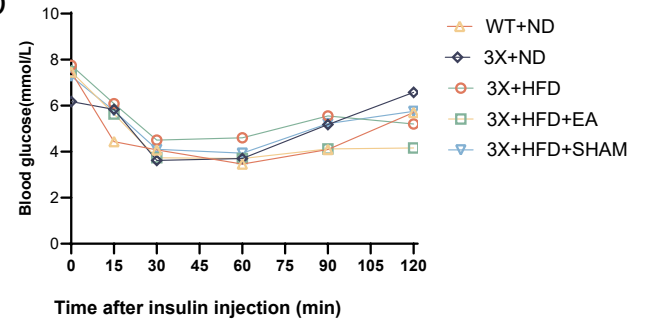

Supplement: Supplementary file 1 — FIGURE S1. EA‐ST36 promotes glucose tolerance in 3XTg mice fed with high‐fat diet. (A) Body weight gain (g) was measured weekly during 16 weeks in wild type (WT) mice fed with normal diet (ND) or high‐fat diet (HFD) and those that received EA treatment or sham EA treatment: WT fed with a normal diet (WT + ND) or 3XTg mice fed with a normal diet (3xTg + ND) or 3XTg mice fed with a high‐fat diet (3xT + HFD). Data are shown as mean ± SEM, n = 10–11. Statistical analysis was performed by two‐way ANOVA with repeated measures followed by post hoc Bonferroni’s multiple comparisons test. (B) Blood glucose levels were measured at several time points following insulin administration during the insulin resistance test (IRT) of WT or 3XTg mice 11 months after of being fed with ND or HFD alone or treated with EA. (C) Blood glucose during intraperitoneal glucose tolerance test (GTT) of WT or mice 11 months after of being fed with ND or HFD alone or treated with EA. (D) Bar graph (lower panel) represents the area under the curve (AUC). Data are shown as mean ± SEM, n = 10 ~ 11 for all the groups. *p < 0.05 versus 3XTg + HFD group analyzed by one‐way ANOVA followed by Tukey’s multiple comparisons test. FIGURE S2. Sham‐EA does not reduce FL‐APP, tau aggregates, or NLRP3–ASC complexes in HFD‐fed 3xTg‐AD mice. (A) Representative western blots of hippocampal (HI) proteins, showing levels of full‐length APP (FL‐APP), CTF‐β/α, total tau, and phosphorylated tau at Ser202/Thr205 (AT8). (B) Quantified data are presented as mean ± SEM. *p < 0.05 versus 3xTg + HFAD (female, n = 6), analyzed by Unpaired t‐test. (C) Representative western blots showed the levels of NLRP3, IL1B, caspase‐1 (CASP1) and ASC in the HI of mice from each group. (D) Data are quantified as mean ± SEM (female, n = 6). *p < 0.05, ns (p > 0.05) versus 3XTg + HFD group analyzed by Unpaired t‐test. FIGURE S3. Viral delivery and survival rate assessment postinjection. (A) Representative image showing hippocampal targeting aft [file CNS-31-e70497-s001.zip › cns70497-sup-0001-FigureS1@FigureS1.pdf]

A

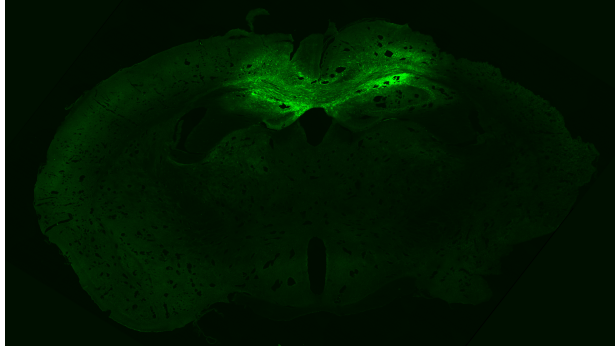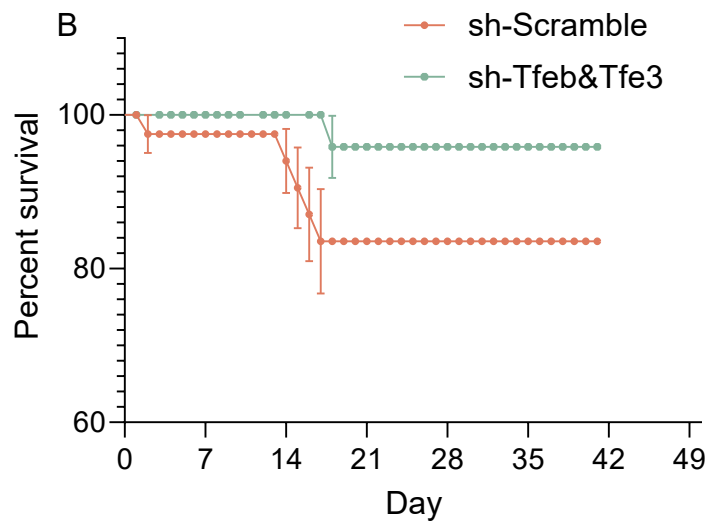

Supplementary Figure1

Supplement: Supplementary file 1 — FIGURE S1. EA‐ST36 promotes glucose tolerance in 3XTg mice fed with high‐fat diet. (A) Body weight gain (g) was measured weekly during 16 weeks in wild type (WT) mice fed with normal diet (ND) or high‐fat diet (HFD) and those that received EA treatment or sham EA treatment: WT fed with a normal diet (WT + ND) or 3XTg mice fed with a normal diet (3xTg + ND) or 3XTg mice fed with a high‐fat diet (3xT + HFD). Data are shown as mean ± SEM, n = 10–11. Statistical analysis was performed by two‐way ANOVA with repeated measures followed by post hoc Bonferroni’s multiple comparisons test. (B) Blood glucose levels were measured at several time points following insulin administration during the insulin resistance test (IRT) of WT or 3XTg mice 11 months after of being fed with ND or HFD alone or treated with EA. (C) Blood glucose during intraperitoneal glucose tolerance test (GTT) of WT or mice 11 months after of being fed with ND or HFD alone or treated with EA. (D) Bar graph (lower panel) represents the area under the curve (AUC). Data are shown as mean ± SEM, n = 10 ~ 11 for all the groups. *p < 0.05 versus 3XTg + HFD group analyzed by one‐way ANOVA followed by Tukey’s multiple comparisons test. FIGURE S2. Sham‐EA does not reduce FL‐APP, tau aggregates, or NLRP3–ASC complexes in HFD‐fed 3xTg‐AD mice. (A) Representative western blots of hippocampal (HI) proteins, showing levels of full‐length APP (FL‐APP), CTF‐β/α, total tau, and phosphorylated tau at Ser202/Thr205 (AT8). (B) Quantified data are presented as mean ± SEM. *p < 0.05 versus 3xTg + HFAD (female, n = 6), analyzed by Unpaired t‐test. (C) Representative western blots showed the levels of NLRP3, IL1B, caspase‐1 (CASP1) and ASC in the HI of mice from each group. (D) Data are quantified as mean ± SEM (female, n = 6). *p < 0.05, ns (p > 0.05) versus 3XTg + HFD group analyzed by Unpaired t‐test. FIGURE S3. Viral delivery and survival rate assessment postinjection. (A) Representative image showing hippocampal targeting aft [file CNS-31-e70497-s001.zip › cns70497-sup-0002-FigureS1@FigureS3.pdf]
